# Plasma p-Tau217 and SPECT-Based eZIS in Mild Cognitive Impairment: Concordance Analysis with Validation in an Amyloid PET Sub-Cohort

**DOI:** 10.3390/diagnostics16172702

**Published:** 2026-08-24

**Authors:** I-Lun Huang, Hiroshi Matsuda, Ya-Tang Pai, Ming-Chyi Pai

**Affiliations:** 1 School of Medicine, College of Medicine, National Cheng Kung University, Tainan 701, Taiwan; i54136355@gs.ncku.edu.tw; 2 Clinical Imaging Center for Healthcare, Nippon Medical School, Tokyo 113-0022, Japan; mhiroshilab@gmail.com; 3 Department of Neurology, Chang Gung Memorial Hospital, Linkou Medical Center, Taoyuan 333, Taiwan; tompizza0@gmail.com; 4 Division of Behavioral Neurology, Department of Neurology, National Cheng Kung University Hospital, College of Medicine, National Cheng Kung University, Tainan 701, Taiwan; 5 Alzheimer’s Disease Research Center, National Cheng Kung University Hospital, Tainan 704, Taiwan; 6 Institute of Gerontology, College of Medicine, National Cheng Kung University, Tainan 701, Taiwan

**Keywords:** Alzheimer’s disease, mild cognitive impairment, plasma p-Tau217, amyloid PET, eZIS, cerebral perfusion SPECT, plasma biomarkers, neurodegeneration

## Abstract

(1) **Background/Objectives**: Blood-based biomarkers have emerged as practical tools for identifying Alzheimer’s disease (AD) pathology in patients with mild cognitive impairment (MCI). Among them, plasma phosphorylated Tau217 (p-Tau217) demonstrates strong associations with cerebral amyloid deposition. In parallel, the easy Z-score Imaging System (eZIS), a quantitative brain perfusion SPECT analysis tool, has been widely used to detect characteristic AD-related hypoperfusion patterns. Although both measures reflect distinct AD processes, the relationship between plasma p-Tau217 and eZIS in MCI remains unclear. (2) **Methods**: This retrospective study included 62 patients with MCI who underwent plasma p-Tau217 testing and brain perfusion SPECT with eZIS analysis. Associations between plasma p-Tau217 and the three eZIS indices (severity, extent, and ratio) were evaluated. Exploratory subgroup analyses were performed using a previously reported plasma p-Tau217 threshold of 0.63 pg/mL. In addition, a validation sub-cohort of 21 participants who underwent plasma p-Tau217 testing, eZIS, and amyloid PET was analyzed to assess concordance with cerebral amyloid pathology. (3) **Results**: Among the three eZIS indices, severity demonstrated the highest sensitivity relative to elevated plasma p-Tau217 levels. However, all eZIS indices showed limited discriminative performance. Optimal eZIS cutoff values derived from the present cohort were higher than previously reported thresholds. In the amyloid PET-validated sub-cohort, plasma p-Tau217 demonstrated closer concordance with amyloid positivity than any individual eZIS parameter. The reduced performance of eZIS appeared to be associated with advanced age, substantial vascular burden, white matter lesions, and cerebral atrophy. (4) **Conclusions**: Plasma p-Tau217 showed a stronger association with cerebral amyloid pathology than eZIS indices in this elderly MCI cohort. Nevertheless, eZIS may provide complementary information regarding downstream neurodegenerative and cerebrovascular processes that are not directly captured by plasma biomarkers. This integrated approach highlights plasma p-Tau217 as a primary screening tool for amyloid pathology to guide disease-modifying therapies (DMTs), alongside eZIS for tracking follow-up mixed co-pathologies.

## 1. Introduction

The recent advent of disease-modifying therapies (DMTs) has fundamentally shifted the clinical paradigm of Alzheimer’s disease (AD), making early and accurate diagnosis paramount, especially at the stage of mild cognitive impairment (MCI) [1,2]. Because the therapeutic benefits of these interventions diminish markedly once high tau burden accumulates and irreversible neurodegeneration occurs, utilizing reliable biomarkers to detect incipient pathological changes is now essential [3].

Although amyloid and tau positron emission tomography (PET) and cerebrospinal fluid (CSF) biomarkers are currently considered reference standards for the in vivo assessment of AD pathology, their high costs and limited availability create significant barriers to broader clinical accessibility and early intervention. Consequently, single-photon emission computed tomography (SPECT) has emerged as a more practical, widely available, and affordable alternative. SPECT facilitates AD diagnosis by detecting regional cerebral hypoperfusion in AD-signature areas, such as the temporoparietal junction, posterior cingulate cortex, and precuneus. Historically, the clinical utility of SPECT was hindered by its reliance on subjective visual interpretation. To address this, the easy Z-score Imaging System (eZIS), introduced by Matsuda et al. in 2007, provided an objective and quantitative method for evaluating hypoperfusion [4,5,6]. Today, it remains widely available in many countries and continues to play an important role in dementia evaluation. The clinical application of eZIS has been extensively validated for distinguishing very early-stage AD, predicting the progression from MCI to dementia, and differentiating among other neurodegenerative disorders [7,8,9,10,11,12,13,14,15].

To detect early pathological changes more efficiently, plasma biomarkers have shown tremendous clinical potential [16]. This is particularly relevant in Asian populations, where cultural reluctance regarding spinal procedures often hinders the widespread application of cerebrospinal fluid (CSF) biomarkers, highlighting the critical need for blood-based alternatives [17]. Standalone plasma p-Tau217 assays have demonstrated high concordance with established amyloid biomarkers (amyloid PET and CSF Aβ42/40) and strong discriminative ability in preclinical AD, offering lower costs and technical barriers compared to ratio-based tests [18,19,20,21,22,23,24,25,26,27,28,29,30,31].

While the robust correlation between plasma p-Tau217 and amyloid PET is well-documented, and the relationship between eZIS and amyloid PET has been independently established [7,8,9], the direct diagnostic concordance between plasma p-Tau217 and eZIS remains an unexplored gap in the literature. Clarifying this relationship is essential for translating research findings into practical clinical diagnostic criteria. Furthermore, little is known regarding how these biomarkers behave in clinically heterogeneous MCI populations characterized by advanced age, white matter disease, brain atrophy, and vascular comorbidities [32].

**Figure 1:**
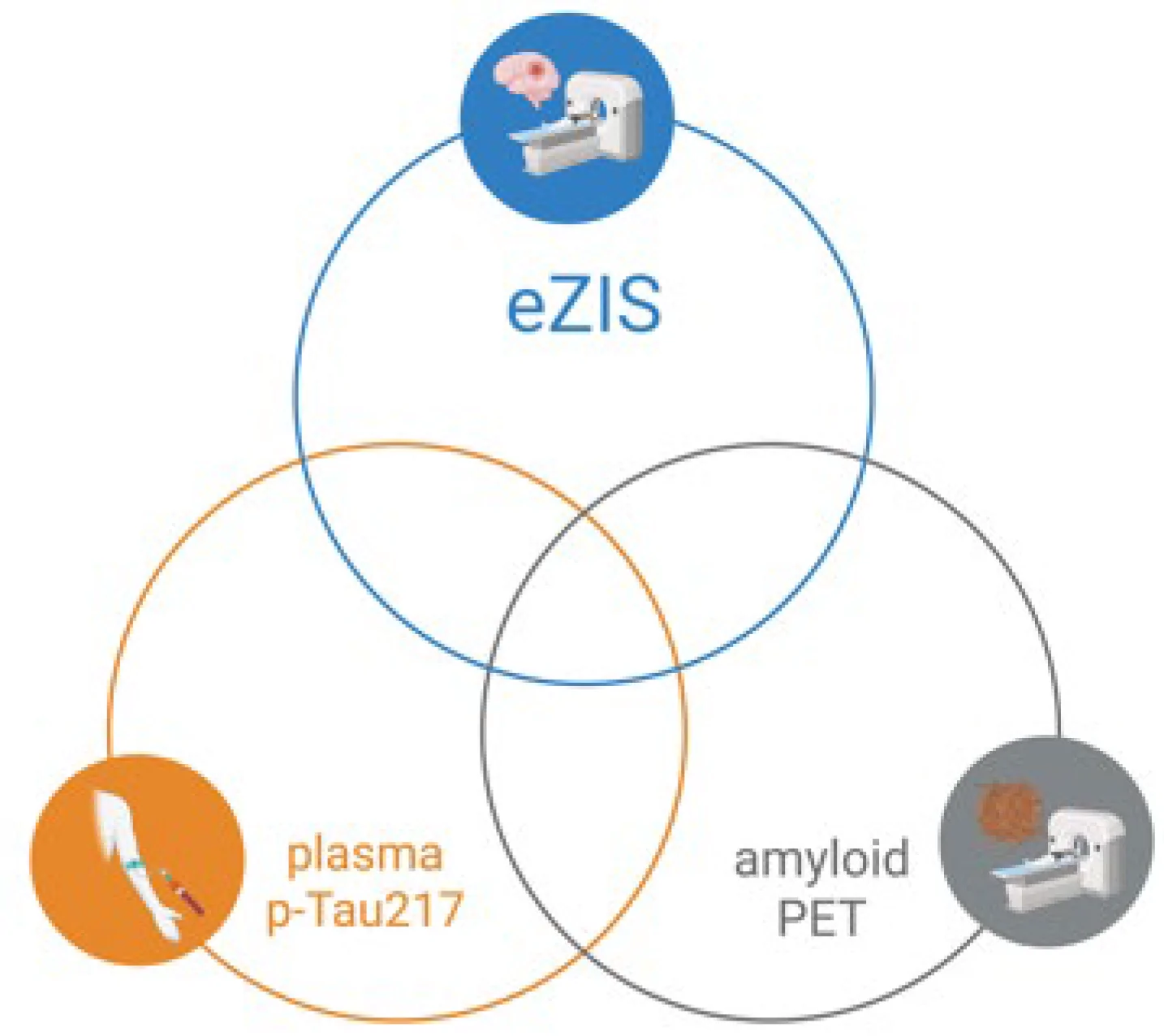
Schematic representation of the study design and research focus (created in https://BioRender.com).

Therefore, we conducted an exploratory head-to-head comparison of plasma p-Tau217 and eZIS in patients with MCI to investigate their relationship and diagnostic concordance. To ensure the reliability of the correlations between these blood-based biomarkers and brain imaging characteristics, all assessments were conducted within a strictly limited time interval. Additionally, we evaluated a unique validation sub-cohort that underwent plasma p-Tau217 testing, eZIS analysis, and amyloid PET imaging, allowing for a direct comparison of these biomarkers against cerebral amyloid pathology. Through this multimodal approach, we sought to clarify the complementary and distinct contributions of blood-based and imaging-based biomarkers in the assessment of MCI (see Figure 1).

## 2. Materials and Methods

### 2.1. Subjects and Research Procedure

This retrospective study recruited individuals with MCI who presented at the memory clinic of National Cheng Kung University Hospital (NCKUH). The clinical diagnosis of MCI was established by a senior behavioral neurologist.

Initially, the study design did not impose a strict temporal limitation between blood sampling for plasma p-Tau217 and the SPECT (eZIS) imaging, resulting in testing intervals extending up to eight years in some cases. However, preliminary evaluations revealed that such extended gaps introduced profound heterogeneity into the dataset. As established in the literature, changes in regional cerebral blood flow, as reflected by eZIS parameters, are inherently nonlinear [33,34], and the accumulation rate of plasma p-Tau217 varies significantly across different stages of disease progression [20]. Consequently, these extended intervals severely compromised the diagnostic concordance and distributional stability between the two modalities. To balance sample size with methodological rigor and minimize confounding effects associated with divergent disease progression trajectories, a strict temporal restriction was enforced. Ultimately, the inclusion criteria for the overall cohort required: (1) a Clinical Dementia Rating (CDR) [35] score of 0.5, and (2) a temporal interval of less than two years between the plasma p-Tau217 assay and SPECT imaging.

To further investigate the diagnostic concordance against the established gold standard, we evaluated a highly characterized multimodal validation sub-cohort comprising participants who additionally underwent amyloid PET imaging. For this specific subset (*n* = 21), the temporal restriction between the blood test and SPECT was relaxed to maximize sample inclusion. Because amyloid PET serves as the definitive pathological anchor, it provided a robust objective basis for the head-to-head comparison with plasma p-Tau217 and eZIS, thereby mitigating potential biases introduced by the extended time interval. Detailed demographic and interval characteristics for this sub-cohort are elaborated in Section 3.

**Figure 2:**
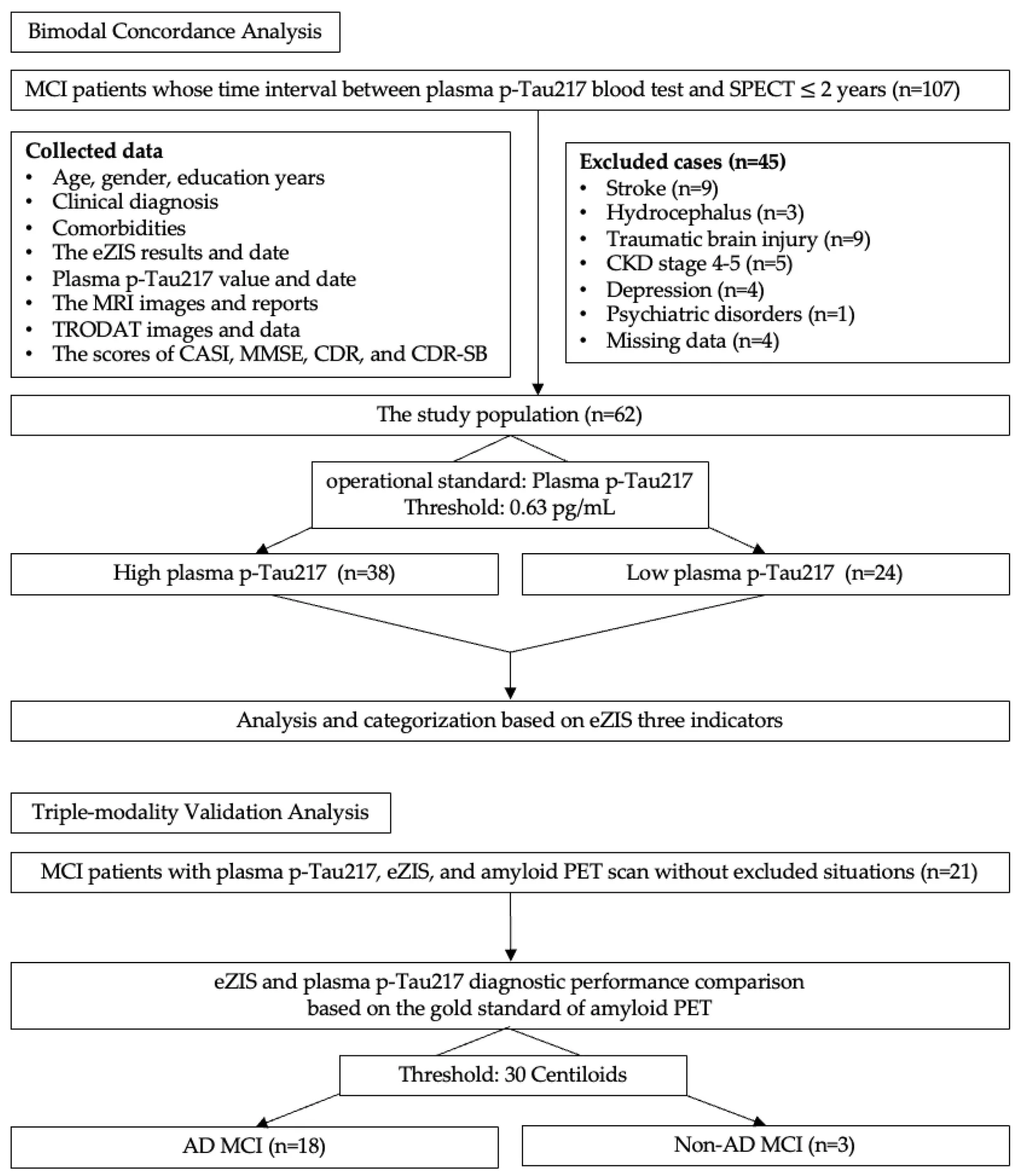
The participant selection and flowchart of this study.

General exclusion criteria for all participants included a history of stroke, hydrocephalus, traumatic brain injury, severe chronic kidney disease (stages 4–5), and major psychiatric disorders, including clinical depression. The detailed participant selection process and study workflow are illustrated in Figure 2.

### 2.2. Clinical Diagnosis

A diagnosis of MCI was made according to Petersen’s criteria: (1) memory complaint, preferably corroborated by an informant; (2) impaired memory function for age and education; (3) preserved general cognitive function; (4) intact activities of daily living; (5) not demented [36].

### 2.3. Examination Tools

#### 2.3.1. Cognitive Assessments

We collected various cognitive assessment scores, including the Cognitive Abilities Screening Instrument (CASI) [37], Mini-Mental Status Examination (MMSE) [38], Clinical Dementia Rating (CDR), and Clinical Dementia Rating-Sum of Boxes (CDR-SB) [35].

#### 2.3.2. Brain Perfusion SPECT and eZIS Analysis

Brain perfusion SPECT imaging was performed approximately 20 min after the intravenous administration of 740 MBq (20 mCi) of Tc-99m ethyl cysteinate dimer (ECD). Image acquisition was performed using a dual-head gamma camera equipped with low-energy high-resolution collimators. Regional cerebral blood flow abnormalities were analyzed using the easy Z-score Imaging System (eZIS), a voxel-based analytical platform developed for the objective quantification of AD-associated hypoperfusion patterns. Three predefined eZIS indices were calculated:

1. Severity: Severity represents the mean positive Z-score within the predefined AD-specific volume of interest (VOI) and reflects the magnitude of regional cerebral blood flow reduction.
2. Extent: Extent represents the percentage of voxels within the AD-specific VOI demonstrating significant hypoperfusion (Z-score > 2).
3. Ratio: Ratio represents the proportion of significantly hypoperfused voxels within the AD-specific VOI relative to those within the entire brain and reflects the specificity of hypoperfusion distribution.

Accuracies for discrimination between healthy controls and patients with very early AD were 85%, 86%, and 80%, respectively. Cutoff values for discrimination were 1.19, 14.2%, and 2.22 for severity, extent, and ratio, respectively. These thresholds were used for exploratory analyses only [4,5,13,14,15].

#### 2.3.3. Plasma p-Tau 217 Measurements

Plasma p-Tau217 levels were quantified using the ALZpath p-Tau217 Simoa assay on a Simoa analyzer (Quanterix, Billerica, MA, USA). To account for the diurnal variation of plasma p-Tau217 reported in previous studies, blood samples were collected during morning clinic hours whenever possible [39,40]. Patients were stratified into high- and low-level groups based on a plasma p-Tau217 operational cutoff value of 0.63 pg/mL [41,42,43].

#### 2.3.4. Amyloid PET Imaging

As this was a single-center study, all amyloid PET and eZIS examinations were performed using standardized protocols within the Department of Nuclear Medicine at National Cheng Kung University Hospital (NCKUH). A subset of participants underwent amyloid PET imaging as part of their clinical evaluation. Following the intravenous administration of 300 MBq of F-18 florbetaben, PET acquisition was performed approximately 90 min post-injection using a Biograph mCT Flow PET/CT scanner (Siemens Healthineers, Erlangen, Germany). Quantitative image analysis was performed using the Centiloid (CL) scale. Based on contemporary recommendations, a Centiloid value of 30 was used to define amyloid positivity [3].

#### 2.3.5. MRI Assessment

Brain MRI examinations were reviewed for the presence and severity of white matter hyperintensities. Periventricular and deep white matter hyperintensities were visually graded according to the Fazekas scale (Grade 0: absent; Grade 1: punctate; Grade 2: beginning confluence; Grade 3: large confluent) [44,45,46]. The images were independently evaluated by two raters (author I.-L.H. and a senior researcher). Across the 59 patients with available MRI data (totaling 118 images), inter-rater reliability was highly consistent, demonstrating an absolute agreement of 94.9% (112/118 images).

#### 2.3.6. TRODAT Imaging

A subset of participants underwent Tc-99m TRODAT-1 SPECT imaging as part of routine clinical evaluation. Following the intravenous administration of 925 MBq (25 mCi) Tc-99m TRODAT-1, SPECT imaging was performed approximately 4 h after injection. The following quantitative measures were recorded: the right striatal TRODAT-1 binding ratio, the left striatal TRODAT-1 binding ratio, and the striatal asymmetry index. Given the limited number of available examinations, the TRODAT findings were analyzed descriptively.

### 2.4. Statistical Analysis

All statistical analyses were performed using Prism version 10 (GraphPad Software, Boston, MA, USA) and SPSS Statistics version 32.0.0.0 (IBM Corp., Armonk, NY, USA).

Continuous variables were summarized as the mean ± standard deviation or median with interquartile range, as appropriate. Categorical variables were summarized as counts and percentages. The Mann–Whitney U test and Fisher’s exact test were used for continuous and categorical variables, respectively. A two-tailed *p*-value < 0.05 was considered statistically significant.

The linear regression model was adjusted for sex, age, and years of education to control for potential confounding effects. A two-tailed *p*-value < 0.05 was considered statistically significant.

Further analysis can be separated into three parts. First, we evaluated the diagnostic performance of each indicator by calculating its sensitivity, specificity, positive predictive value (PPV), and negative predictive value (NPV). Second, receiver operating characteristic (ROC) curve analysis was performed, and the Youden index was calculated to determine the optimal diagnostic cutoff values for our study cohort. These cohort-specific cutoffs were subsequently compared against established reference thresholds. Third, we calculated the overall diagnostic accuracy of each indicator, defined as the proportion of correctly classified subjects (true positives and true negatives) out of the total sample [47].

### 2.5. Disclosure of Generative Artificial Intelligence (GenAI) Use

Gemini 3.1 Pro was utilized for English grammar correction and language refinement.

## 3. Results

### 3.1. Clinical Features

We identified 62 MCI patients who met the research criteria, including 38 patients with high plasma p-Tau217 level and 24 patients with low plasma p-Tau217 level, defined by the threshold of 0.63 pg/mL for plasma p-Tau217. The demographic and clinical characteristics are listed in Table 1. Due to the complexity of the demographic data table, a simplified distribution of participants across various categories, stratified by plasma p-Tau217 and eZIS three indicators, is presented in Appendix A, Table A1, Table A2 and Table A3.

Additionally, we identified 21 MCI patients who met the criteria for the multimodal validation sub-cohort, comprising 18 mild cognitive impairment due to Alzheimer’s disease (AD MCI) and 3 mild cognitive impairment due to non-Alzheimer’s disease (non-AD MCI) cases, as defined by an amyloid PET Centiloid (CL) threshold of 30. The demographic and clinical characteristics of this specific sub-cohort are detailed in Table 2. Compared with the overall cohort, this subgroup demonstrated a high prevalence of amyloid positivity, consistent with referral bias in clinical amyloid PET utilization. The mean Centiloid value was markedly higher among the amyloid-positive participants than among amyloid-negative participants.

### 3.2. Outcomes

#### 3.2.1. The Relation Between the eZIS Three Indicators and Plasma p-Tau217

The relationship between the plasma p-Tau217 concentrations and the three eZIS indices was evaluated across the entire cohort. Participants with higher plasma p-Tau217 concentrations tended to demonstrate higher eZIS severity, extent, and ratio values. However, substantial overlap was observed between groups, indicating considerable heterogeneity within the MCI population. Among the three eZIS indices, severity demonstrated the strongest numerical association with elevated plasma p-Tau217 levels. Although the mean severity value was higher in the high p-Tau217 group compared to the low p-Tau217 group, this difference did not reach statistical significance, even after adjusting for sex and years of education in a linear regression model. Notably, within these models, age emerged as a significant independent predictor for both severity and extent. Similarly, extent and ratio values were numerically higher in the high p-Tau217 group but exhibited broad distributions and overlapping interquartile ranges. These findings suggest that while cerebral perfusion abnormalities and plasma p-Tau217 levels may be biologically linked, these two biomarkers likely capture distinct pathophysiological aspects of disease progression.

Exploratory diagnostic performance analyses were performed using previously established eZIS reference thresholds. Among the three eZIS indices, severity demonstrated the highest sensitivity relative to elevated plasma p-Tau217 levels. However, while severity showed the greatest sensitivity, specificity remained modest across all three indicators, reflecting a limited ability to definitively confirm elevated p-Tau217 status based solely on eZIS abnormalities. Overall, concordance between plasma p-Tau217 status and eZIS abnormalities was limited, suggesting that these biomarkers do not provide interchangeable information. Diagnostic performance metrics are summarized in Table 3.

**Figure 3:**
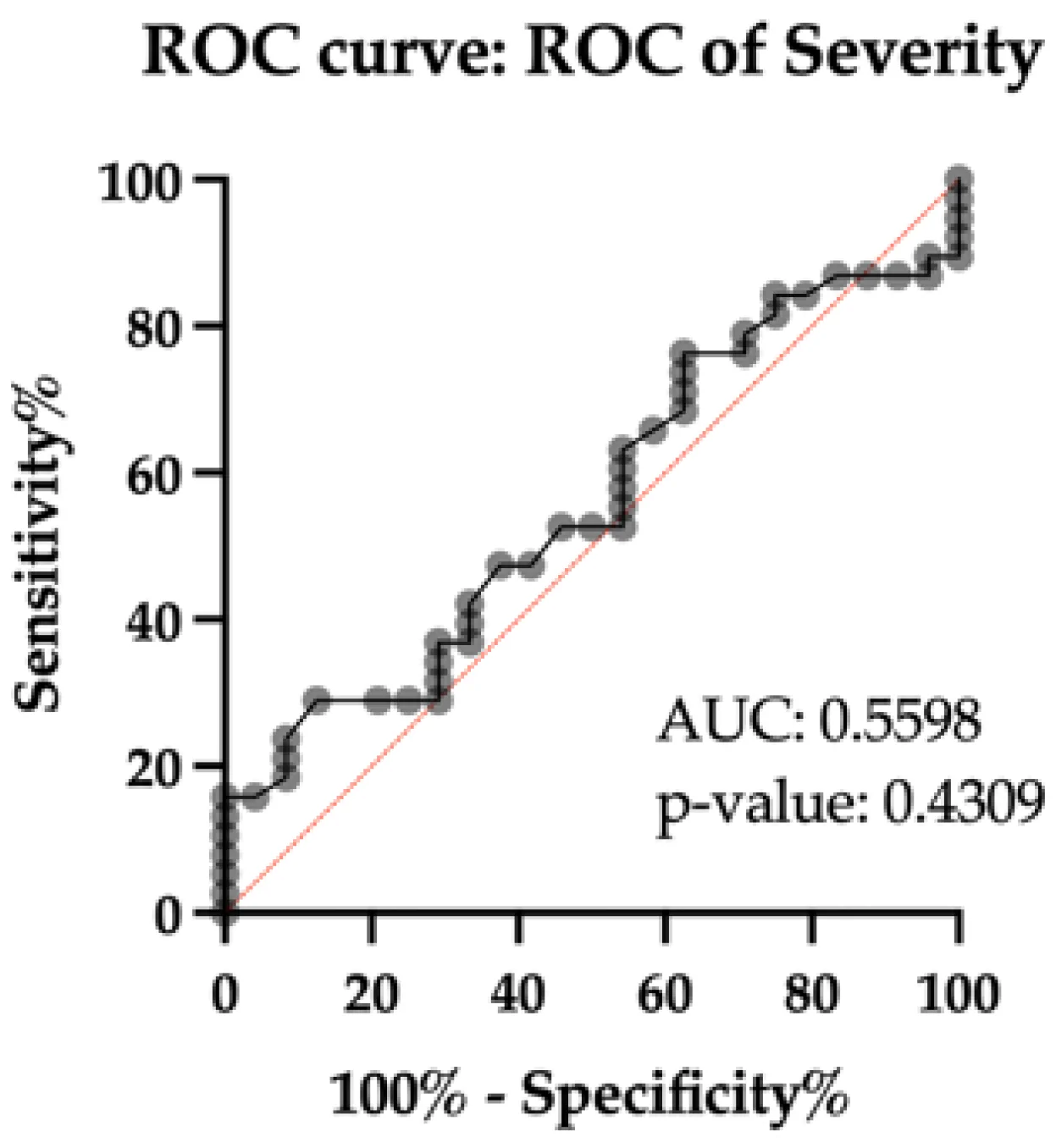
The ROC curve of severity. The diagonal red line serves as the line of no-discrimination.

**Figure 4:**
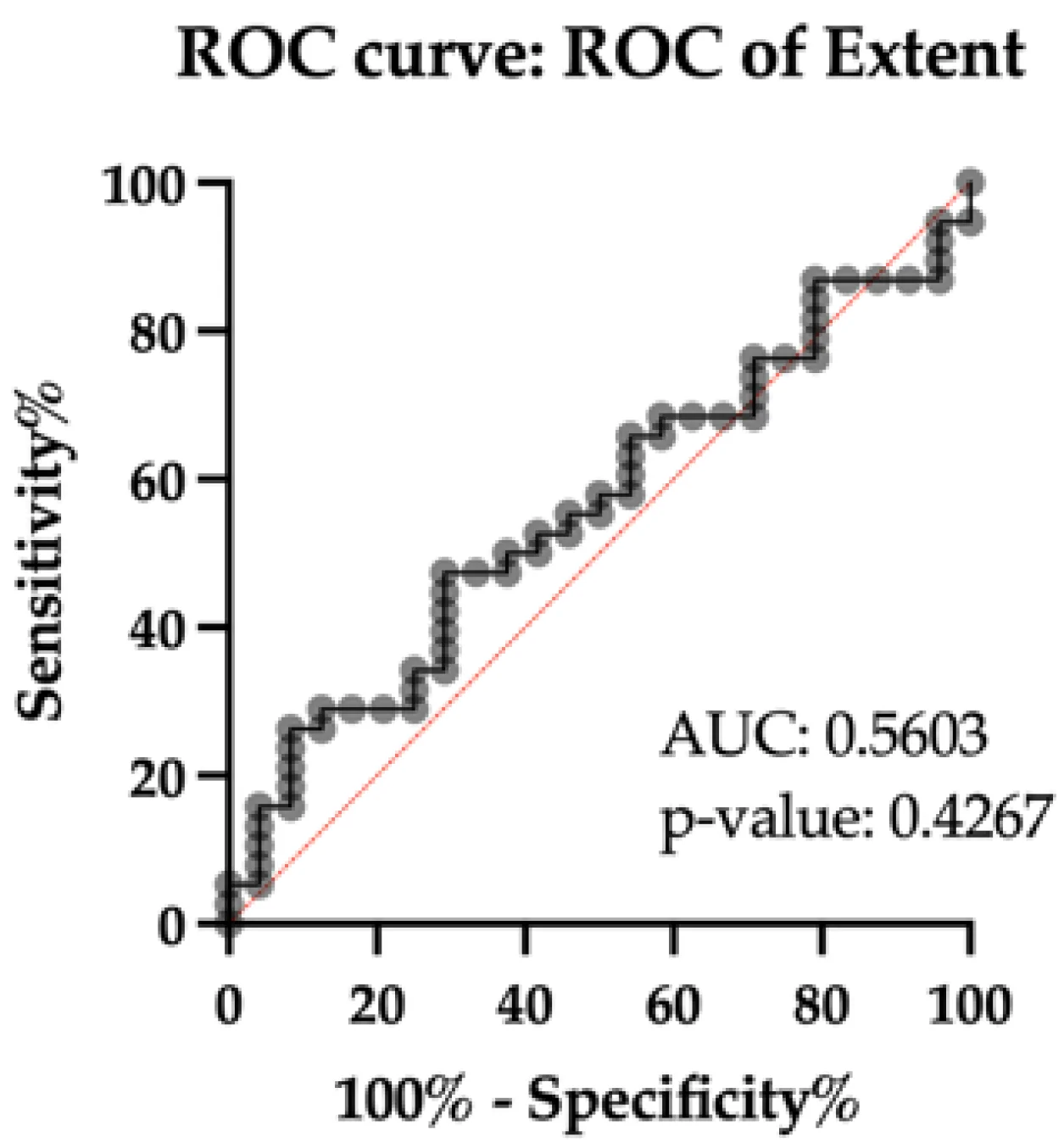
The ROC curve of extent. The diagonal red line serves as the line of no-discrimination.

**Figure 5:**
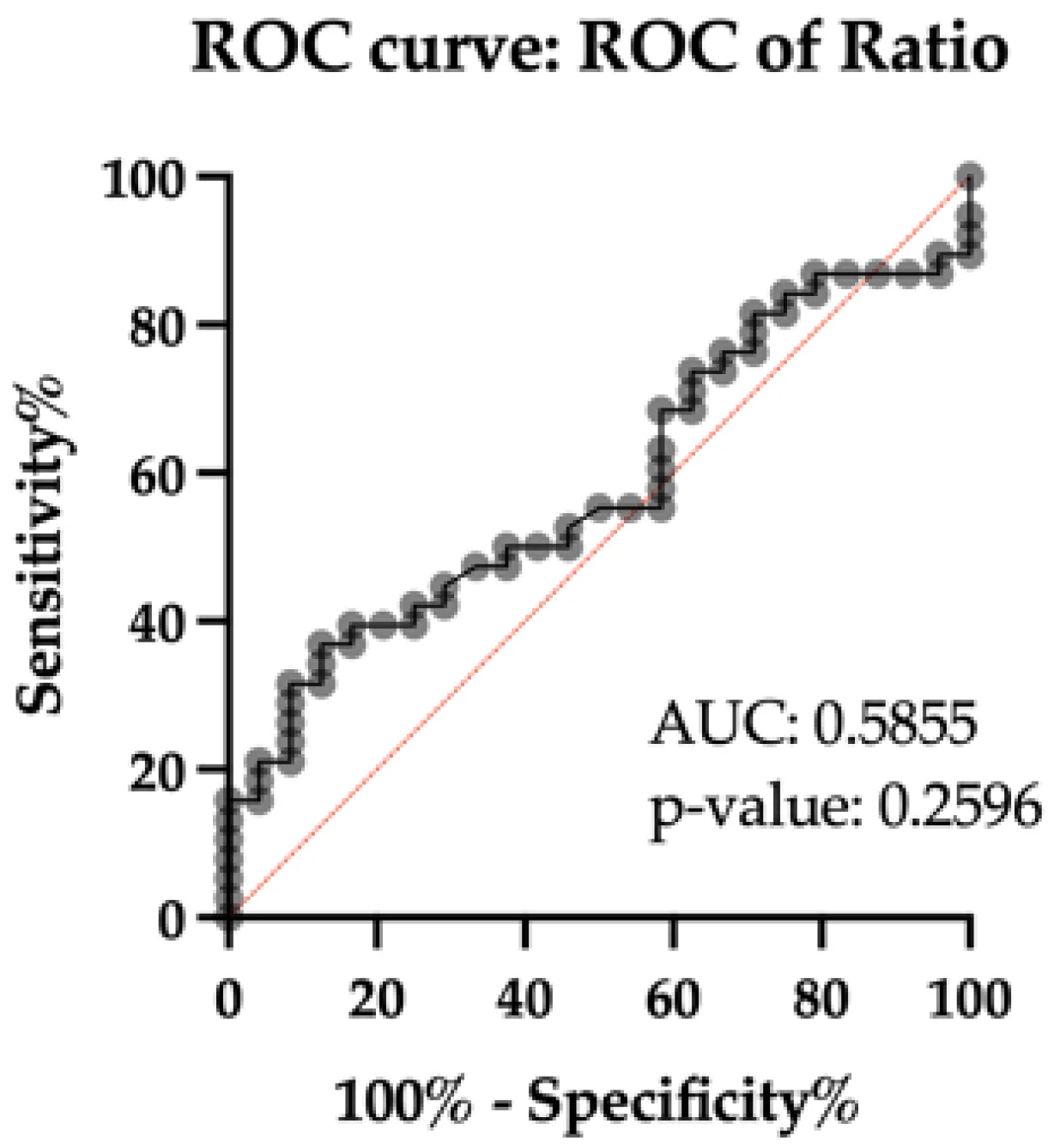
The ROC curve of ratio. The diagonal red line serves as the line of no-discrimination.

Subsequent receiver operating characteristic (ROC) analyses using the Youden index were performed to establish cohort-specific eZIS thresholds, yielding an area under curve (AUC) of approximately 0.57, as shown in Figure 3, Figure 4 and Figure 5. The newly optimized cutoffs for severity, extent, and ratio were 1.78, 21.46, and 3.805, respectively, which were notably higher than the original reference values summarized in Table 4. Since we excluded stroke patients, the relatively high values of the three indices are most likely caused by chronic vascular pathology, as indicated by the MRI reports and Fazekas scale results.

#### 3.2.2. The Amyloid PET Positive Probability, eZIS, and Plasma p-Tau217

The availability of complete data across all three diagnostic platforms represents a rare clinical scenario, enabling a precise head-to-head validation against cerebral amyloid pathology. Within this multimodal validation cohort, 14 patients had a time interval of less than two years between plasma p-Tau217 sampling and eZIS imaging, while 7 had an interval exceeding two years. This extended timeframe was permitted to maximize the inclusion of these high-value data. Using amyloid PET as the absolute reference standard, plasma p-Tau217 demonstrated a diagnostic performance superior to that of eZIS. Specifically, the sensitivity of plasma p-Tau217 reached 94.4%, whereas the highest sensitivity among the three eZIS indicators was achieved by the severity index at 72.2%. Full diagnostic performance metrics are detailed in Table 5.

Although plasma p-Tau217 showed stronger agreement with amyloid PET positivity, all diagnostic estimates should be interpreted cautiously because of the limited number of amyloid-negative participants. Notably, several amyloid-positive participants exhibited only mild abnormalities on eZIS, whereas some participants with elevated eZIS indices demonstrated relatively low amyloid burden. These observations support the notion that plasma p-Tau217 and cerebral perfusion abnormalities reflect related but biologically distinct processes.

#### 3.2.3. Summary of Main Findings

Three principal findings emerged from this study. First, plasma p-Tau217 and eZIS indices demonstrated only modest concordance in a clinically heterogeneous MCI population. Second, optimal eZIS thresholds in this elderly cohort were higher than the previously reported reference values, suggesting an influence of vascular pathology, white matter disease, and cerebral atrophy on perfusion-based biomarkers. Third, within the amyloid PET validation cohort, plasma p-Tau217 demonstrated closer concordance with cerebral amyloid pathology than any individual eZIS parameter. Collectively, these findings suggest that plasma p-Tau217 and eZIS provide complementary rather than interchangeable information regarding biological processes occurring along the Alzheimer disease continuum.

## 4. Discussion

Based on the three key findings, we present the following discussion.

### 4.1. Plasma p-Tau217 and eZIS Reflect Different Biological Processes

A key observation of this study is that plasma p-Tau217 and eZIS should not be viewed as competing biomarkers. Rather, they appear to reflect different biological processes occurring along the AD continuum.

According to the contemporary AT(N) framework, biomarkers may be classified according to the pathological processes they represent, including amyloid deposition (A), tau pathology (T), and neurodegeneration (N) [3]. Plasma p-Tau217 has consistently demonstrated strong associations with both amyloid and tau pathology and is increasingly regarded as a blood-based surrogate marker of AD pathology. In contrast, cerebral perfusion abnormalities measured by eZIS are likely to reflect downstream functional consequences of neurodegeneration, synaptic dysfunction, and network disconnection.

To interpret these results, we propose a conceptual visualization using two opposing arrows. Plasma p-Tau217 acts as an arrow pointing directly to amyloid pathology, representing the upstream core of AD. Conversely, eZIS highlights hypoperfusion, reflecting downstream manifestations. Given these opposing positions in the pathological cascade, we infer that complex co-pathologies exert a more profound influence on the presentation of eZIS.

This biological distinction may explain the relatively modest concordance observed between plasma p-Tau217 and eZIS in the present study. Amyloid accumulation may precede substantial reductions in cerebral blood flow by several years, whereas perfusion abnormalities may become more prominent during later stages characterized by neuronal injury and functional impairment. Consequently, discordance between these biomarkers should not necessarily be interpreted as diagnostic disagreement but rather as evidence that they capture different stages of disease progression.

### 4.2. Why Was eZIS Performance Lower than Expected?

Previous studies have reported a favorable diagnostic performance of eZIS for distinguishing early AD from cognitively normal individuals and for predicting progression from MCI to dementia. In contrast, the present study demonstrated relatively limited discrimination by all three eZIS indices.

Several factors may explain this finding. Primarily, the present cohort was substantially older than the populations evaluated in the original eZIS validation studies. With a mean age exceeding 75 years, these patients presented with highly heterogeneous clinical profiles. Notably, a subset of participants who underwent TRODAT imaging exhibited relatively low binding ratios, suggesting the presence of mixed neurodegenerative pathologies rather than pure AD (see Appendix A, Table A4). Additionally, cerebral perfusion is inherently influenced by multiple age-related processes beyond AD pathology. Common vascular comorbidities in this older cohort, such as chronic hypertension, small vessel disease, and white matter hyperintensities, may potentially contribute to regional reductions in cerebral blood flow. Consequently, these perfusion abnormalities could render SPECT less specific for cerebral amyloid deposition compared to molecular biomarkers directly related to AD [48,49]. Finally, structural brain changes further confound eZIS measurements through partial volume effects. Although the system provides objective quantification of regional cerebral blood flow, significant cortical volume loss (cerebral atrophy) prevalent in elderly populations can artificially reduce measured perfusion values. Together, these structural and vascular confounders increase variability and limit the overall diagnostic specificity of perfusion-based biomarkers in real-world clinical settings.

These considerations are supported by our ROC analyses, which suggested optimal eZIS thresholds substantially higher than the previously reported reference values. This observation suggests that standard cutoff values derived from younger or less vascularly burdened populations may not be directly applicable to elderly memory-clinic populations.

Furthermore, as contemporary diagnostic frameworks increasingly incorporate concepts of brain resilience and cognitive reserve, it is understood that clinical manifestations of cognitive decline are often delayed in individuals with higher educational and socio-behavioral engagement [50,51,52]. As a result, these patients frequently present at an older age, harboring a significantly higher burden of age-related comorbidities, such as cerebrovascular pathology and brain atrophy. This demographic reality may explain the attenuated diagnostic performance of eZIS and underscores the need for age- and comorbidity-adjusted diagnostic approaches in real-world clinical settings.

### 4.3. Validation Against Amyloid PET

The validation sub-cohort provided an opportunity to compare plasma p-Tau217 and eZIS directly against cerebral amyloid pathology.

Within this subgroup, plasma p-Tau217 demonstrated stronger concordance with amyloid PET positivity than any individual eZIS parameter. This finding is consistent with a growing body of literature demonstrating close associations between plasma p-Tau217 and amyloid PET across the AD continuum.

From a biological perspective, this result is not unexpected. Plasma p-Tau217 is closely linked to molecular events occurring during amyloid-associated tau dysregulation, whereas cerebral perfusion abnormalities represent functional consequences that may be influenced by multiple additional factors.

Furthermore, previous large-scale studies have reported that the PPV of plasma p-Tau217 for predicting amyloid PET positivity can reach approximately 93% [20,53]. In contrast, our head-to-head comparison revealed that the highest PPV between the plasma p-Tau217 and eZIS indicators was only 66%. This discrepancy of nearly 30% highlights the significant impact of clinical cofactors. Specifically, complex co-pathologies and varied trajectories of downstream neurodegeneration might independently lead to cerebral hypoperfusion, thereby uncoupling functional imaging results from pure molecular AD pathology.

Importantly, however, several participants demonstrated discordant findings between plasma p-Tau217, eZIS, and amyloid PET. Some amyloid-positive individuals exhibited only mild perfusion abnormalities, whereas others demonstrated substantial perfusion reductions despite relatively modest amyloid burden. Such observations further support the concept that perfusion imaging and blood biomarkers provide complementary information rather than redundant measurements.

Accordingly, our findings should not be interpreted as evidence that plasma p-Tau217 can replace functional neuroimaging. Instead, plasma biomarkers and perfusion imaging appear to contribute distinct information regarding pathological burden, neurodegeneration, and cerebrovascular status.

### 4.4. Clinical Implications

The present findings yield several practical implications for clinical practice.

Foremost, plasma p-Tau217 appears highly suitable as an initial screening biomarker for identifying individuals who harbor underlying AD pathology. Compared with amyloid PET or cerebrospinal fluid analyses, blood-based testing offers substantial advantages in terms of accessibility, cost-effectiveness, and patient acceptance. Thus, blood-based biomarkers represent a robust, non-invasive, and practical modality for aiding AD diagnosis and prognostic staging, particularly serving as a primary gatekeeper to identify patients suitable for DMTs. Furthermore, recent longitudinal clock models utilizing plasma p-Tau217 have demonstrated that older individuals experience a markedly shorter interval between p-Tau217 positivity and the onset of clinical symptoms [54]. As these authors emphasized, age-related co-pathologies, such as cerebrovascular disease, not only confound functional imaging results but also actively accelerate the progression of dementia in p-Tau217-positive elderly patients.

In addition, despite its weaker association with specific amyloid pathology, eZIS retains significant clinical utility. Perfusion abnormalities reflect the downstream functional impact of the disease on cerebral networks, capturing contributions from both neurodegenerative and vascular mechanisms. Consequently, the diagnostic value of eZIS may be particularly pronounced in early-onset AD (EOAD), where patients typically present with a purer AD pathological profile and a minimal burden of vascular co-pathologies. In these cases of relatively pure AD, eZIS can effectively delineate the characteristic AD-specific hypoperfusion patterns. Conversely, in patients with mixed etiologies, elevated or atypical eZIS indices can serve as a critical warning sign, indicating the presence of overlapping pathologies and the need for further diagnostic workup. Importantly, in the context of DMTs, eZIS may hold potential utility for longitudinal follow-up, although this warrants further long-term investigation. It could hypothetically serve as a valuable tool to track downstream functional changes and evaluate whether a suboptimal clinical response to therapy is driven by the progression of mixed co-pathologies.

Ultimately, the integration of blood-based biomarkers and functional imaging provides a more comprehensive assessment than either modality alone. While plasma biomarkers can accurately detect the presence of molecular AD pathology, perfusion imaging characterizes its downstream functional consequences and the compounding effects of coexisting cerebrovascular disease. This multimodal approach will become increasingly vital as DMTs are integrated into routine clinical practice, where the precise biological characterization and staging of individual patients are essential for determining treatment eligibility.

### 4.5. Limitations

Several limitations should be acknowledged. First, the overall sample size was relatively modest. This is primarily because eZIS, plasma p-Tau217, and amyloid PET are not routinely required in current standard clinical diagnostic procedures. Furthermore, strict temporal inclusion criteria were applied to minimize disease progression-related confounding between plasma sampling and SPECT acquisition, which further limited the sample size. Second, the amyloid PET validation cohort was small and highly imbalanced, consisting of 18 amyloid-positive and only 3 amyloid-negative participants. Consequently, estimates of specificity and negative predictive value (NPV) should be interpreted cautiously, despite the head-to-head design of the study. Third, the retrospective single-center design may limit generalizability to other populations. Fourth, the study population consisted primarily of elderly patients with substantial vascular burden and white matter disease. Although this characteristic reflects real-world clinical practice, the findings may differ in younger populations or in patients with more biologically pure AD pathology. Finally, the present study focused on cerebral perfusion SPECT and plasma p-Tau217. Future prospective investigations incorporating additional multimodal biomarkers and functional clinical metrics, such as immersive spatial navigation assessments and the correlation of focal cerebral hypoperfusion with biomarker profiles, are warranted to provide a more comprehensive understanding of biomarker interactions and clinical trajectories across the AD continuum.

## 5. Conclusions

In this exploratory analysis of an elderly MCI cohort, plasma p-Tau217 demonstrated closer concordance with cerebral amyloid pathology than eZIS-derived perfusion measures. However, the modest agreement observed between these biomarkers suggests that they capture different biological aspects of disease progression.

Rather than representing competing diagnostic tools, plasma p-Tau217 and eZIS appear to provide complementary information regarding amyloid pathology, neurodegeneration, and cerebrovascular burden. Plasma p-Tau217 can guide initial treatment eligibility, while eZIS provides essential longitudinal insights into mixed etiologies and functional disease trajectories. Future prospective studies are needed to determine whether integrating both blood-based and neuroimaging biomarkers can facilitate a more comprehensive evaluation of heterogeneous MCI populations. Such integration may better capture the transition from MCI to AD and improve biological characterization across the AD continuum.

## Figures and Tables

**Table 1:** Demographic and clinical characteristics.

	Low Plasma p-Tau217 (*n* = 24)	High Plasma p-Tau217 (*n* = 38)	*p*-Value
Demographic data			
Age when p-Tau217 performed (mean, [IQR], SD)	75.29 [70.25, 82.75] 7.147	75.55 [72.00, 80.25] 6.616	0.7491
Education, years (mean, [IQR], SD)	8.583 [6, 12] 4.323	9.684 [6, 14] 5.353	0.4300
Gender (male) (*n*, percentage)	9 (37.5%)	10 (26.32%)	0.4030
Comorbidities			
Hypertension	10 (41.7%)	18 (47.4%)	0.7945
Dyslipidemia	7 (29.2%)	7(18.4%)	0.3626
Diabetes mellitus	11 (45.8%)	8 (21.1%)	0.0509
Hearing impairment	6 (25.0%)	8 (21.1%)	0.7617
Coronary artery disease	5 (20.8%)	6(15.8%)	0.7362
Insomnia	9 (37.5%)	9 (23.7%)	0.2652
Microbleed	2 (8.33%)	3 (7.89%)	1.000
Cognitive assessments			
CDR-SB (mean, [IQR], SD)	1.583 [0.625, 2.375] 0.9286	1.961 [1.500, 2.500] 0.8958	0.1348
MMSE (mean, [IQR], SD)	22.67 [20.00, 25.75] 3.306	21.37 [18.75, 23.25] 3.071	0.1549
CASI			
Total score (mean, [IQR], SD)	75.04 [68.25, 84.50] 9.989	70.00 [66.00, 76.25] 9.623	0.0757
Remote memory (mean, [IQR], SD)	9.708 [10.00, 10.00] 0.9079	9.737 [10.00, 10.00] 0.6851	0.9186
Recent memory **** (mean, [IQR], SD)	6.708 [4.250, 8.750] 2.596	3.737 [2.000, 5.000] 2.127	<0.0001
Attention (mean, [IQR], SD)	7.000 [6.000, 8.000] 1.142	6.895 [6.000, 8.000] 1.226	0.7970
Mental manipulation (mean, [IQR], SD)	7.083 [6.000, 10.00] 2.636	7.921 [7.000, 10.00] 2.148	0.1976
Orientation * (mean, [IQR], SD)	14.54 [13.00, 17.75] 3.401	12.26 [9.000, 17.00] 3.950	0.0113
Abstract thinking (mean, [IQR], SD)	6.667 [6.000, 8.000] 1.308	7.053 [6.000, 8.000] 1.064	0.3076
Language (mean, [IQR], SD)	9.417 [9.000, 10.00] 0.6539	9.132 [9.000, 10.00] 1.044	0.4534
Drawing (mean, [IQR], SD)	8.708 [8.000, 10.00] 1.989	8.342 [8.000, 10.00] 2.831	0.7552
Verbal fluency (mean, [IQR], SD)	5.042 [4.000, 6.000] 1.459	4.921 [4.000, 6.000] 1.634	0.7678
The eZIS indicators			
Severity (mean, [IQR], SD)	1.367 [1.030, 1.698] 0.3952	1.515 [1.183, 1.838] 0.6338	0.4358
Extent (mean, [IQR], SD)	18.52 [8.825, 26.15] 13.92	23.82 [8.963, 36.88] 19.49	0.4317
Ratio (mean, [IQR], SD)	2.319 [1.120, 3.288] 1.456	3.089 [1.510, 4.425] 2.162	0.2633
Severity ≥ 1.19 (*n*, percentage)	15 (62.5%)	29 (76.3%)	0.2652
Extent ≥ 14.2 (*n*, percentage)	13 (54.2%)	24 (63.2%)	0.5989
Ratio ≥ 2.22 (*n*, percentage)	14 (58.3%)	21 (55.3%)	1.000
Zero abnormal indicator (*n*, percentage)	9 (37.5%)	9 (23.7%)	0.2652
One abnormal indicator (*n*, percentage)	0 (0.00%)	3 (7.90%)	0.2766
Two abnormal indicators (*n*, percentage)	3 (12.5%)	7 (18.4%)	0.7272
Three abnormal indicators (*n*, percentage)	12 (50.0%)	19 (50.0%)	1.000
More than two abnormal indicators (*n*, percentage)	15 (62.5%)	26 (68.4%)	0.7837
Fazekas scale (PWMH, DWMH) (*n*, percentage)			
(0–1, 0–1)	11 (45.8%)	21 (55.3%)	0.4368
(0–1, 2–3)	0 (0.00%)	0 (0.00%)	
(2–3, 0–1)	3 (12.5%)	3 (7.90%)	
(2–3, 2–3)	8 (33.3%)	7 (18.4%)	
No MRI data	2 (8.33%)	7 (18.4%)	
Biomarker			
Plasma p-Tau217 value **** (mean, [IQR], SD)	0.3631 [0.2270, 0.4973] 0.1590	1.236 [0.8393, 1.612] 0.5331	<0.0001

Note. IQR: interquartile range; CASI: Cognitive Abilities Screening Instrument; MMSE: Mini-mental State Examination; CDR: Clinical Dementia Rating; SB: sum of boxes; eZIS: easy Z-score imaging system; Plasma p-Tau217: plasma phosphorylated tau217; PWMH: periventricular white matter hyperintensity DWMH: deep white matter hyperintensity **** *p* < 0.0001 * *p* < 0.05.

**Table 2:** Demographic and clinical characteristics of the 21 participants who underwent all three diagnostic evaluations (eZIS, plasma p-Tau217, and amyloid PET).

	Non-AD MCI (*n* = 3)	AD MCI (*n* = 18)	*p*-Value
Demographic data			
Age when p-Tau217 performed (mean, [IQR], SD)	76.33 [70.00, 81.00] 5.686	72.39 [69.00, 75.50] 4.925	0.2856
Education, years * (mean, [IQR], SD)	17.33 [14.00, 22.00] 4.163	10.89 [6.00, 14.50] 4.364	0.0489
Gender (male) (*n*, percentage)	2 (66.7%)	5 (27.8%)	0.2474
Comorbidities			
Hypertension	2 (66.7%)	10 (55.6%)	1.0000
Dyslipidemia *	2 (66.7%)	1 (5.56%)	0.0414
Diabetes mellitus *	2 (66.7%)	0 (0.00%)	0.0143
Hearing impairment	0 (0.00%)	3 (16.7%)	1.0000
Coronary artery disease	0 (0.00%)	3 (16.7%)	1.0000
Insomnia	0 (0.00%)	6 (33.3%)	0.5263
Microbleed	0 (0.00%)	1 (5.56%)	1.0000
Cognitive assessments			
CDR-SB (mean, [IQR], SD)	2.000 [0.500, 4.000] 1.803	1.972 [1.375, 2.625] 0.8484	0.7992
MMSE * (mean, [IQR], SD)	26.00 [24.00, 29.00] 2.646	22.78 [21.75, 24.25] 1.833	0.0451
CASI			
Total score * (mean, [IQR], SD)	83.33 [76.00, 91.00] 7.506	74.22 [71.25, 76.50] 4.930	0.0383
Remote memory (mean, [IQR], SD)	10.00 [10.00, 10.00] 10.00	9.944 [10.00, 10.00] 0.2357	>0.9999
Recent memory * (mean, [IQR], SD)	8.677 [7.000, 11.00] 2.082	4.222 [3.000, 5.250] 2.157	0.0180
Attention (mean, [IQR], SD)	8.000 [8.000, 8.000] 0.000	7.278 [6.750, 8.000] 1.074	0.4263
Mental manipulation (mean, [IQR], SD)	8.667 [6.000, 10.00] 2.309	8.722 [8.000, 10.00] 1.565	0.9173
Orientation (mean, [IQR], SD)	16.00 [13.00, 18.00] 2.646	12.00 [9.750, 13.00] 3.308	0.0714
Abstract thinking (mean, [IQR], SD)	7.333 [7.000, 8.000] 0.5774	7.111 [6.750, 8.000] 1.023	0.7060
Language (mean, [IQR], SD)	9.333 [9.000, 10.00] 0.5774	9.611 [9.000, 10.00] 0.7775	0.5211
Drawing (mean, [IQR], SD)	9.333 [9.000, 10.00] 0.5774	9.667 [10.00, 10.00] 1.188	0.0797
Verbal fluency (mean, [IQR], SD)	6.000 [5.000, 7.000] 1.000	5.889 [4.750, 7.000] 1.530	>0.9999
The eZIS indicators			
Severity (mean, [IQR], SD)	1.510 [1.200, 1.950] 0.3915	1.364 [1.123, 1.488] 0.4095	0.7248
Extent (mean, [IQR], SD)	16.95 [9.090, 22.59] 7.020	19.51 [8.160, 22.22] 15.34	0.8977
Ratio (mean, [IQR], SD)	2.413 [1.280, 4.420] 1.743	2.931 [1.613, 4.018] 2.037	0.5338
Severity ≥ 1.19 (*n*, percentage)	3 (100%)	13 (72.2%)	0.5489
Extent ≥ 14.2 (*n*, percentage)	2 (66.7%)	12 (66.7%)	1.000
Ratio ≥ 2.22 (*n*, percentage)	1 (33.3%)	10 (55.6%)	0.5865
Zero abnormal indicator (*n*, percentage)	0 (0.00%)	5 (27.8%)	0.5489
One abnormal indicator (*n*, percentage)	1 (33.3%)	0 (0.00%)	0.1429
Two abnormal indicators (*n*, percentage)	1 (33.3%)	4 (22.2%)	1.0000
Three abnormal indicators (*n*, percentage)	1 (33.3%)	9 (50.0%)	1.0000
More than two abnormal indicators (*n*, percentage)	2 (66.7%)	13 (72.2%)	1.0000
Fazekas scale (PWMH, DWMH) (*n*, percentage)			
(0–1, 0–1)	1 (33.3%)	11 (61.1%)	0.6526
(0–1, 2–3)	0 (0.00%)	1 (5.56%)	
(2–3, 0–1)	0 (0.00%)	2 (11.1%)	
(2–3, 2–3)	1 (33.3%)	4 (22.2%)	
No MRI data	1 (33.3%)	0 (0.00%)	
**Biomarker**			
Plasma p-Tau217 value (mean, [IQR], SD)	0.9327 [0.6450, 1.280] 0.3217	1.272 [0.8375, 1.822] 0.6146	0.4707
Centiloid scale			
Centiloid scale value ** (mean, [IQR], SD)	0.8333 [−7.900, 7.600] 7.935	88.12 [54.35, 104.6] 42.30	0.0015

Note. AD-MCI: mild cognitive impairment due to Alzheimer’s disease; non-AD MCI: mild cognitive impairment due to non-Alzheimer’s disease; IQR: interquartile range; CASI: Cognitive Abilities Screening Instrument; MMSE: Mini-mental State Examination; CDR: Clinical Dementia Rating; SB: sum of boxes; eZIS: easy Z-score imaging system; Plasma p-Tau217: plasma phosphorylated tau217; PWMH: periventricular white matter hyperintensity; DWMH: deep white matter hyperintensity. ** *p* < 0.01 * *p* < 0.05.

**Table 3:** Diagnostic performance of the three eZIS indicators.

	Severity	Extent	Ratio
Sensitivity	76.3%	63.2%	55.3%
Specificity	37.5%	41.7%	45.8%
PPV	66.0%	64.9%	60.0%
NPV	50.0%	44.0%	37.0%
Diagnostic accuracy	61.3%	56.5%	50.0%

Note. PPV: positive predictive value; NPV: negative predictive value.

**Table 4:** Changes in the cutoff thresholds for the three eZIS indicators.

	Severity	Extent	Ratio
Original cutoffs	1.19	14.2	2.22
New cutoffs	1.78	21.46	3.805

**Table 5:** Diagnostic performance of plasma p-Tau217 and three eZIS indicators.

	Plasma p-Tau217	Severity	Extent	Ratio
Sensitivity	94.4% (17/18)	72.2% (13/18)	66.7% (12/18)	55.6% (10/18)
Specificity	0% (0/3)	0% (0/3)	33.3% (1/3)	66.7% (2/3)
PPV	85.0% (17/20)	81.25% (13/16)	85.7% (12/14)	90.9% (10/11)
NPV	0% (0/1)	0% (0/5)	14.3% (1/7)	20.0% (2/10)
Diagnostic accuracy	81.0% (17/21)	61.9% (13/21)	61.9% (13/21)	57.1% (12/21)

Note. A value of 0% for specificity and NPV indicates the absence of true negatives; specifically, no patient in this cohort tested negative for both amyloid PET and the respective index (plasma p-Tau217 or eZIS severity). PPV: positive predictive value; NPV: negative predictive value.

## Data Availability

The data presented in this study are available on request from the corresponding author.

## Abbreviations

The following abbreviations are used in this manuscript:

AD	Alzheimer’s disease
eZIS	Easy Z-Score Imaging System
MCI	Mild cognitive impairment
p-Tau217	Phosphorylated Tau217
PET	Positron emission tomography
rCBF	Regional cerebral blood flow
SPECT	Single-photon emission computed tomography
VOI	Volume of interest
DMTs	Disease-modifying therapies

## Appendix A

**Table A1:** Distribution of 62 participants according to the eZIS severity and plasma p-Tau217 results.

	Plasma p-Tau217 ≥ 0.63 pg/mL	Plasma p-Tau217 < 0.63 pg/mL
**Severity** ≥ **1.19**	29	15
**Severity** < **1.19**	9	9

**Table A2:** Distribution of 62 participants according to the eZIS extent and plasma p-Tau217 results.

	Plasma p-Tau217 ≥ 0.63 pg/mL	Plasma p-Tau217 < 0.63 pg/mL
**Extent** ≥ **14.2**	24	13
**Extent** < **14.2**	14	11

**Table A3:** Distribution of 62 participants according to the eZIS ratio and plasma p-Tau217 results.

	Plasma p-Tau217 ≥ 0.63 pg/mL	Plasma p-Tau217 < 0.63 pg/mL
**Ratio** ≥ **2.22**	21	14
**Ratio** < **2.22**	17	10

**Table A4:** Statistical values of the TRODAT-1 SPECT imaging parameters in the 12 patients.

Right Striatal TRODAT-1 Binding Ratio	Left Striatal TRODAT-1 Binding Ratio	Striatal Asymmetry Index
0.4212 [0.3470, 0.4558] 0.1561	0.4774 [0.3618, 0.5613] 0.1843	15.20 [4.750, 22.43] 11.32

Note. Data are presented as (mean, [IQR], SD).
